# Supplementary material for: In Vitro and In Vivo Inhibition of MATE1 by Tyrosine Kinase Inhibitors
Source: Pharmaceutics. 2021 Nov 25;13(12):2004. doi: 10.3390/pharmaceutics13122004 (PMC8707461; doi:10.3390/pharmaceutics13122004)
Supplement: Supplementary file 1 [file pharmaceutics-13-02004-s001.zip › pharmaceutics-1444451-supplementary.pdf]

pharmaceutics

MDPI

Supplementary Materials: In Vitro and In Vivo Inhibition of MATE1 by Tyrosine Kinase Inhibitors

Muhammad Erfan Uddin, Zahra Talebi1, Sijie Chen, Yan Jin, Alice A. Gibson, Anne M. Noonan, Xiaolin Cheng, Shuiying Hu and Alex Sparreboom

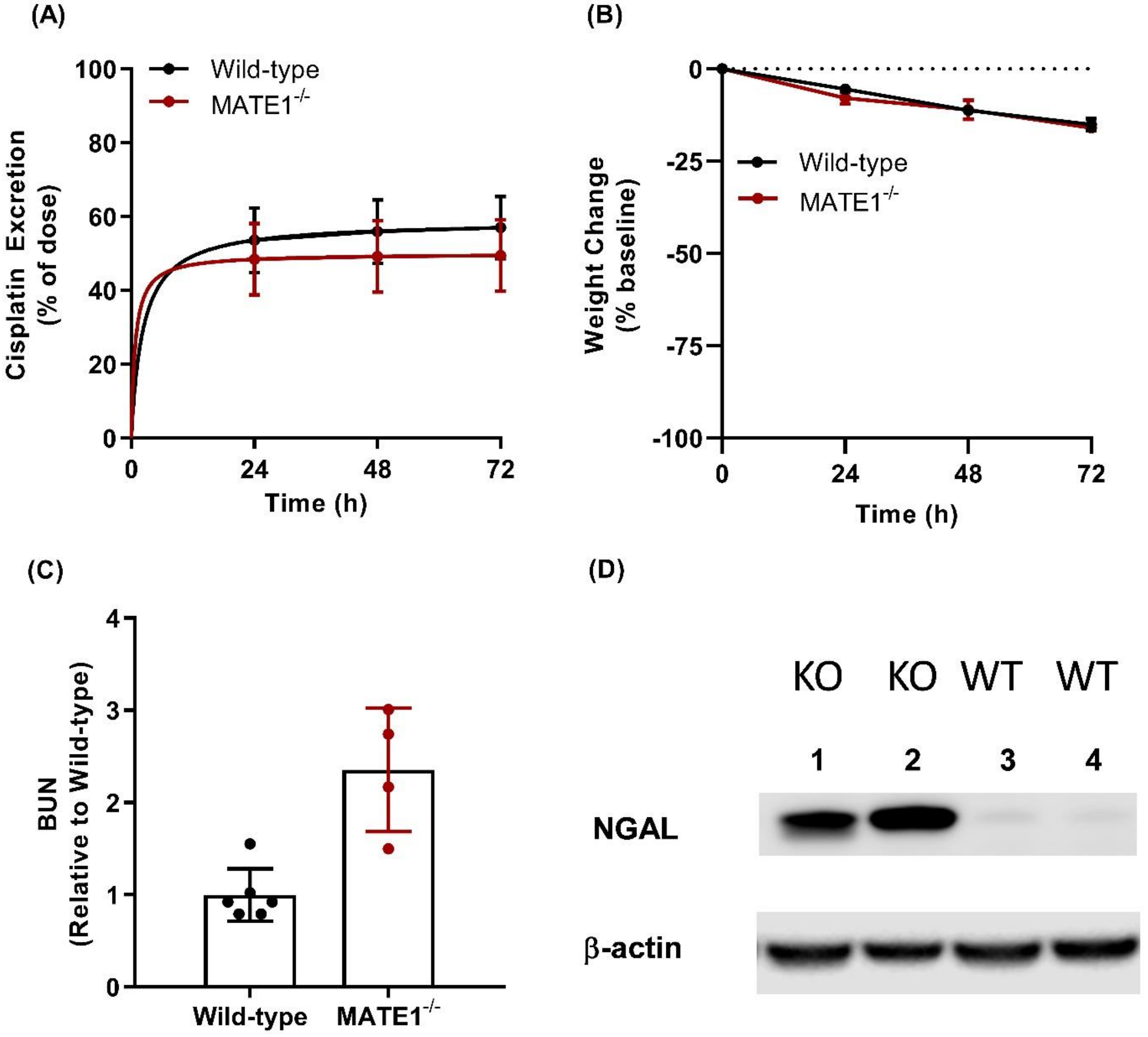

Figure S1. Influence of MATE1 deficiency on cisplatin disposition. Urinary excretion **(A)**, weight loss **(B)**, BUN **(C)**, and the kidney damage marker NGAL **(D)** were analyzed in wild-type mice and MATE1-deficient mice (n=5 each) following the administration of a single dose of cisplatin (15 mg/kg, i.p.). Data represent mean ± SEM.

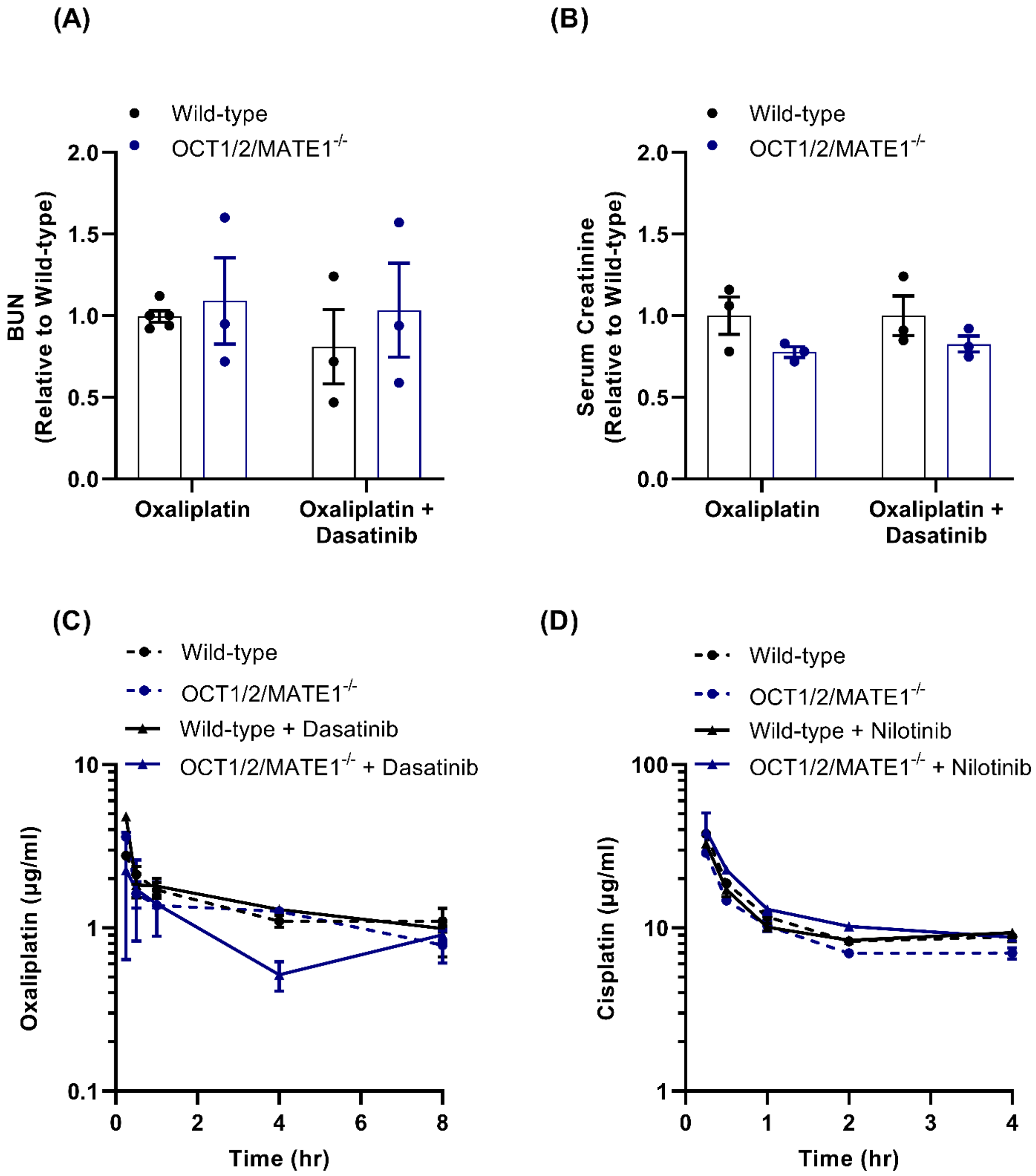

Figure S2. Influence of TKI pre-treatment on oxaliplatin and cisplatin disposition. (A) BUN and **(B)** Serum creatinine were assessed in wild-type mice and OCT1/2/MATE1-deficient mice (n=3 each) following the administration of a single dose of oxaliplatin (10 mg/kg, i.p.) with and without dasatinib pre-treatment (15 mg/kg, p.o.). Data represent mean ± SEM. Plasma concentration time profile of oxaliplatin (10 mg/kg, i.p.) with or without dasatinib pre-treatment (15 mg/kg, p.o.) **(C)** and cisplatin (15 mg/kg, i.p.) with or without nilotinib pre-treatment (10 mg/kg, p.o.) **(D)**. See Methods for details.

Table S1. Serum chemistry parameters in wild-type (WT) and OCT1/2/MATE1-deficient mice (Triple KO) at baseline.

			Male		Female	
**Serum Chemistry**	**Unit**	**Normal Range**	**WT**	**Triple KO**	**WT**	**Triple KO**
**Albumin**	g/dL	2.5-3.9	3.57	3.40	3.03	3.27
**Alkaline Phosphatase**	U/L	23-181	109	113	125	165
**Alanine Aminotransferase**	U/L	16-58	37.0	50.7	50.3	78.2
**Aspartate Aminotransferase**	U/L	36-102	64.3	81.3	73.7	96.0
**Blood Urea Nitrogen**	mg/dL	14-32	21.0	19.7	16.7	18.7
**BUN/Creatinine Ratio**			64.3	69.9	192	65.2
**Calcium**	mg/dL	7.6-10.7	12.0	11.0	12.0	11.1
**Chloride**	mmol/L	103-115	108	117	128	128
**Cholesterol**	mg/dL	74-190	248	252	186	177
**Creatinine**	mg/dL	0.1-0.6	0.34	0.28	0.16	0.28
**Creatinine Kinase**	U/L	110-1609	122	178	126	131
**Gammaglutamyl Transferase**	U/L	0-2	3.67	4.67	4.33	3.33
**Globulins**		1.3-2.8	2.77	2.87	2.73	2.47
**Glucose**	mg/dL	76-222	419	321	371	240
**Potassium**	mmol/L	3.4-5.5	12.9	12.3	11.9	10.6
**Phosphorus**	mg/dL	4.6-9.3	11.7	10.9	11.3	13.0
**Sodium**	mmol/L	146-155	154	161	161	170
**Total Bilirubin**	mg/dL	0.0-0.3	0.67	0.70	0.50	0.43
**Total Protein**	g/dL	4.1-6.4	6.33	6.27	5.77	5.73
**Trigylceride**	mg/dL	66-246	339	383	243	383
**Uric Acid**	mg/dL	3.44-3.71	2.30	2.93	5.20	4.43

Table S2. Publicly available information on TKI-mediated inhibition of MATE1.

Name	Target(s)	First approval and indication	% inhibition at 10 μM	FDA/ EMA	Published literature	IC50	Ref
Acalabrutinib	BTK	2017 mantle cell lymphoma, CLL	43.3	×			
Afatinib	HER2, EGFR	2013 NSCLC with EGFR mutations	89.6		√	< 10 μM	1
Alectinib	ALK	2014 NSCLC with ALK translocations	0				
Avapritinib	PDGFR, KIT	2020 GIST	78.6	√			
Axitinib	VEGFR2	2012 renal cell carcinoma	60.8		√	< 10 μM	1
Baricitinib	JAKs	2017 rheumatoid arthritis	55.5	√			
Binimetinib	MEK, RAF	2018 melanoma	47.6	×		>50 μM	
Bosutinib	BCR-ABL, SRC	2012 CML	99.2		√	< 10 μM	1
Brigatinib	ALK, EGFR	2017 NSCLC with EGFR mutation	95.9	√			
Cabozantinib	VEGFR2, PDGFR, KIT	2012 medullary thyroid cancer	95.8	√	√	< 10 μM	1,2
Capmatinib	MET	2020 NSCLC	99.7	√	√		
Ceritinib	ALK	2014 NSCLC with ALK translocations	96.1	√			
Cobimetinib	MEK	2015 melanoma	83.3				
Crizotinib	ALK, MET	2011 NSCLC with ALK mutation	96.8	√	√	0.34 μM	1,3
Dabrafenib	BRAF	2013 skin cancer	72.1		√		1
Dacomitinib	EGFR	2018 NSCLC with EGFR mutations	91.6				
Dasatinib	BCR-ABL, SRC	2006 CML, ALL	95.4		√	0.84 μM	1,4
Encorafenib	MEK, RAF	2018 melanoma	66.1				
Entrectinib	TRK, ROS, ALK	2019 NSCLC	99.6	√			
Erdafitinib	FGFR	2019 urothelial cancer	97.0	×			
Erlotinib	EGFR	2004 NSCLC, pancreatic cancer	87.0		√	7.93 μM	1,4
Fedratinib	JAK	2019 myelofibrosis	98.6	√	√		5
Fostamatinib	SYK	2018 autoimmune thrombocytopenia	40.2				
Gefitinib	EGFR	2003 NSCLC	91.8		√	1.92 μM	1,3,4
Gilteritinib	FLT3, AXL	2018 AML	96.7	√	√		
Ibrutinib	BTK	2013 mantle cell lymphoma, CLL	91.8				

Imatinib	BCR-ABL, KIT, PDGFR	2001 CML	98.9		√	0.05 µM	1,3,4,6
Lapatinib	EGFR, ERBB2	2007 breast cancer	38.7		×	> 10 µM	1
Larotrectinib	NTRK	2018 cancer with NTRK fusions	21.1	×			
Lenvatinib	VEGFR	2015 thyroid cancer (DTC), kidney cancer	87.7				
Lorlatinib	ALK, ROS	2018 NSCLC	61.8	√			
Midostaurin	FLT3, KIT	2017 AML, mastocytosis	50.0	√			
Neratinib	EGFR	2017 breast cancer HER2	90.9				
Nilotinib	BCR-ABL	2007 CML	95.7		√	3.38 µM	1,4
Nintedanib	VEGFR, PDGFR, FGFR	2014 idiopathic pulmonary fibrosis	95.3				
Olmutinib	EGFR	2016 NSCLC with EGFR mutation	97.7				
Osimertinib	EGFR	2015 NSCLC with EGFR mutation	94.1	√			
Pazopanib	Multiple	2009 renal cell carcinoma, soft tissue sarcomas	90.2		√	< 0.47 µM	1,3
Pemigatinib	FGFR2	2020 cholangiocarcinoma with FGFR2 fusion	80.1	√			
Pexidartinib	CSF1R, KIT	2019 TGCT	75.6	√			
Ponatinib	SRC, BCR-ABL	2012 CML, ALL	84.9		√	< 10 µM	1
Pralsetinib	RET	2020 NSCLC, thyroid cancers	94.5	√			
Radotinib	BCR-ABL, PDGFR	2012 CML	73.0				
Regorafenib	TIE2, PTKs	2012 colorectal cancer, GIST, HCC	72.1		√	< 10 µM	1
Ripretinib	KIT	2020 GIST, Mastocytosis	51.9	√			
Ruxolitinib	JAK	2011 myelofibrosis	63.0		√	< 10 µM	1
Selpercatinib	RET	2020 NSCLC, MTC	97.4	√		0.66 µM	
Sorafenib	Multiple	2005 renal cancer, HCC	83.8		√	1.43 µM	1,3,4,7
Sunitinib	Multiple	2006 renal cancer, GIST	98.0		√	0.28 µM	1,3,4
Tivozanib	VEGFR	2017 RCC	90.3	x			
Tofacitinib	JAKs	2012 rheumatoid arthritis	90.4		√	~ 10 µM	1
Trametinib	MEK	2013 skin cancer	96.1	√		0.06 µM	
Tucatinib	HER2	2020 HER2 breast cancer	96.3	√	√		8

Upadacitinib	JAK	2019 rheumatoid arthritis	77.2	√			
Vandetanib	Multiple	2011 thyroid cancer	99.5		√	< 10 μM	1
Vemurafenib	BRAF	2011 metastatic melanoma	34.1				
Zanubrutinib	BTK	2019 mantle cell lymphoma	94.5				

√ denotes inhibition of MATE1; × denotes no inhibition of MATE1 at clinically-relevant concentrations.

Table S3. Plasma pharmacokinetic parameters of cisplatin and oxaliplatin in mice and a human patient with cancer.*

Treatment	Species	C_{max} (μg/ml)	AUC (h×μg/ml)
**Cisplatin**	Mouse		
Wild-type		45.4 ± 2.04	43.2 ± 2.08
Wild-type + Nilotinib		37.1 ± 1.57	37.6 ± 1.57
OCT1,2/MATE1$^{-/-}$		40.2 ± 2.37	39.3 ± 1.26
OCT1,2/MATE1$^{-/-}$ + Nilotinib		30.6 ± 2.77	32.2 ± 1.20
**Oxaliplatin**	Mouse		
Wild-type		2.76 ± 0.04	10.7 ± 1.00
Wild-type + Dasatinib		4.80 ± 0.01	12.2 ± 0.71
OCT1,2/MATE1$^{-/-}$		3.61 ± 0.18	10.5 ± 0.01
OCT1,2/MATE1$^{-/-}$ + Dasatinib		3.86 ± 0.01	7.53 ± 0.90

**Oxaliplatin**	Human		
Oxaliplain		0.96	11.3
Oxaliplatin + Dasatinib		0.87	11.6

*Cisplatin (15 mg/kg i.p.) and oxaliplatin (10 mg/kg, i.p.) were administered to male wild-type and OCT1/2/MATE1-deficient mice (n=5 each) with and without pretreatment with nilotinib (10 mg/kg, p.o.) or dasatinib (15 mg/kg, p.o.), respectively. The human patient received oxaliplatin as an i.v. infusion at a dose of 85 mg/m^2 with and without dasatinib pre-treatment (100 mg, p.o.).

Abbreviations: C_{max}, peak plasma concentration; AUC, area under the plasma concentration-time curve.

References

1. Sprowl JA, Ong SS, Gibson AA, et al. A phosphotyrosine switch regulates organic cation transporters. Nat Commun. 2016 Mar 16;7:10880.
2. Lacy, S., Hsu, B., Miles, D., Aftab, D., Wang, R., & Nguyen, L. (2015). Metabolism and disposition of cabozantinib in healthy male volunteers and pharmacologic characterization of its major metabolites. *Drug Metabolism and Disposition*, *43*(8), 1190-1207.
3. Omote, S., Matsuoka, N., Arakawa, H., Nakanishi, T., & Tamai, I. (2018). Effect of tyrosine kinase inhibitors on renal handling of creatinine by MATE1. *Scientific reports*, *8*(1), 1-11.
4. Minematsu, T., & Giacomini, K. M. (2011). Interactions of tyrosine kinase inhibitors with organic cation transporters and multidrug and toxic compound extrusion proteins. *Molecular cancer therapeutics*, *10*(3), 531-539.
5. Ogasawara, K., Wood-Horrall, R. N., Thomas, M., Thomas, M., Liu, L., Liu, M., ... & Krishna, G. (2021). Impact of fedratinib on the pharmacokinetics of transporter probe substrates using a cocktail approach. *Cancer Chemotherapy and Pharmacology*, 1-12.
6. Vidal-Petiot, E., Rea, D., Serrano, F., Stehlé, T., Gardin, C., Rousselot, P., ... & Flamant, M. (2016). Imatinib increases serum creatinine by inhibiting its tubular secretion in a reversible fashion in chronic myeloid leukemia. *Clinical Lymphoma Myeloma and Leukemia*, *16*(3), 169-174.
7. Karbownik, A.; Szkutnik-Fiedler, D.; Czyrski, A.; Kostewicz, N.; Kaczmarska, P.; Bekier, M.; Stanisławiak-Rudowicz, J.; Karaźniewicz-Łada, M.; Wolc, A.; Główka, F.; Grześkowiak, E.; Szałek, E. Pharmacokinetic Interaction between Sorafenib and Atorvastatin, and Sorafenib and Metformin in Rats. *Pharmaceutics* **2020**, *12*, 600. https://doi.org/10.3390/pharmaceutics12070600
8. Topletz-Erickson, A. R., Lee, A. J., Mayor, J. G., Rustia, E. L., Abdulrasool, L. I., Wise, A. L., ... & Endres, C. J. (2021). Tucatinib inhibits renal transporters OCT2 and MATE without impacting renal function in healthy subjects. *The Journal of Clinical Pharmacology*, *61*(4), 461-47.
